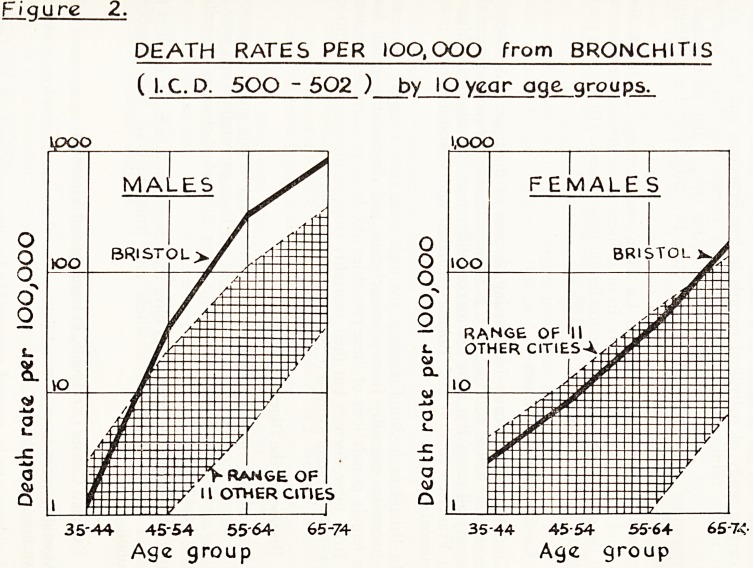# A Comparative Mortality with Special Reference to Bristol

**Published:** 1968-04

**Authors:** R. C. Wofinden

**Affiliations:** Medical Officer of Health, City and County of Bristol; Professor of Public Health, University of Bristol


### A COMPARATIVE MORTALITY STUDY WITH SPECIAL REFERENCE TO BRISTOL

(Inter-American Investigation of Mortality)

BY

R. C. WOFINDEN, M.D., M.R.C.P., D.P.H., D.P.A.

*Medical Officer of Health, City and County of Bristol;*
*Professor of Public Health, University of Bristol.*

### Edward Long Fox's Death

"By the death of Dr. Edward Long Fox which occurred on Friday, March 28th, Bristol loses one of her best loved and most prominent citizens. Dr. Fox's health had been failing for some time prior to the illness which definitely commenced nearly a year ago. Rallying for a time, he persevered in his professional work, preferring death in harness to seeking the complete rest he sorely needed. In July of last year he went to Malvern, returning but little better for the change from which so much was hoped, and from that time to the date of his death he became gradually weaker owing to the increasing development of gouty glycosuria. For many weeks he suffered acutely from neuritis of the sciatic nerve, but towards the close of his illness he was fortunately free from pain."

So ran the opening sentences of Fox's obituary notice in the B.M.J. of April 5th, 1902. Dr. J. E. Shaw who certified his death, ascribed it to diabetes mellitus of 7 years standing and to exhaustion. What was the cause of his death? The two statements, taken together, illustrate a continuing problem for students of mortality statistics—the need for accuracy in the certification of the cause of death although clearly this has to be within the limits of existing medical and scientific knowledge at any moment of time.

The collection of comprehensive and accurate information on the causes of death is a most important step in a nation's struggle for medical progress and improvement in the health of the people.

### Death Certification—History in U.K.

Britain was an early leader in this field. Systematic registration of baptisms, burials and weddings was required by every priest of the Established Church of England from 1538 and elementary analysis of the causes of death was carried out. But John Graunt in his "Observations on the Bills of Mortality" in 1662 was probably the first to demonstrate the great potential value of analysed mortality data as an instrument of medicine. His observations created only a fragmentary and desultory interest for the next century or so. It was not until Dr. William Farr was appointed as "Compiler of Abstracts" to the G.R.O. (formed under the Births and Deaths Registration Act of 1836) that a reasoned need for accuracy in death certification was clearly stated :

"Diseases are more easily prevented than cured, and the first step to their prevention is the discovery of their exciting causes. The registry will show the agency of these causes by numerical facts and measure the intensity of their influence and will collect information on the laws of vitality with the variation in these laws in the two sexes at different ages and the influence of civilization, occupation, locality, seasons and other physical agencies either in generating diseases and inducing death or in improving the public health."

Farr was well aware of the possibility of error in certification and he issued explanatory notes from time to time to all authorised medical practitioners. Some idea of the limitations of medical knowledge (in particular in differentiating disease), and of the incompleteness of the process of registration are shown by the first entries in the Bristol Registrar's Death Register in 1837. The free issue of a book of death certificates to all doctors from 1847 onwards did much to improve the completeness and accuracy of death certification although practitioners were not *compelled* to complete certificates until 1874. It was not until 1904 that suggestions regarding the completion of the certificates were printed as a preface to the official book of forms and in 1927 the Registrar General exercised his new power (under the Birth and Death Registration Act 1926) to prescribe and design the certificate. It was from this date that the attempt was first made to get doctors to think, and certify, in terms of the *underlying*, as well as the *immediate* cause of death. The reason, of course, was to facilitate central classification on a uniform basis and to ensure comparability of data with the passage of time.

It is important for a country to know its leading causes of death, the age groups, sexes, and the social and occupational classes in which they occur. Thereby attention can be focused on problems needing priority. Comparisons over a period of time can give a measure of success or failure in tackling old problems or throw up new ones and regional disparities may highlight local environmental hazards.

### Death Certification—International Comparability

According to the W.H.O., " Efforts to secure international comparability in health statistics began as early as 1851 on the initiative of certain intergovernmental and voluntary organisations ". For proper comparability there must be a " a uniform nomenclature and classification of diseases and causes of death " and this means establishing uniform rules within which countries must operate (in other words standardisation is necessary). International agreements stemmed from the 1948 W.H.O. assembly leading to an official form of death certificate and three official lists (full, with 999 causes; intermediate, with 150 causes; and abbreviated, with 50 causes) for the International Classification of Diseases (I.C.D.). The I.C.D. is periodically revised on an agreed international basis and is currently under review.

### The Inter-American Mortality Survey

There are still many countries in which there is no adequate machinery for registration and certification of births and deaths. But even in the well developed nations the differing standards of training for doctors, fashions and standards in diagnosis and certification, the varying accessibility of patients to medical care, and different levels of development in their health services lead to considerable reservations about the degree of reliability which can be placed on international vital statistics. The Inter-American Investigation of Mortality (1962-64), in which Bristol and all Bristol doctors participated was

### AGE-ADJUSTED MORTALITY RATES AT AGES 15-74 PER 100,000 AT RISK BY MAJO CAUSE GROUPS

Numbers in brackets refer to the code numbers of the International Classification of Diseases 1955 (W.H.O. 1957)

| | Tuberculosis and other Infectious and parasitic diseases (001-138) | | Malignancies (140-205) | | Cardiovascular diseases (330-334, 400-468) | | Respiratory diseases (470-527) | | Diseases of the digestive system (530-587) | | External causes (E800-E999) | |
|---|---|---|---|---|---|---|---|---|---|---|---|---|
| | Male | Female | Male | Female | Male | Female | Male | Female | Male | Female | Male | Female |
| Bogota ... ... ... | 48 | 34 | 115 | 128 | 223 | 232 | 46 | 47 | 53 | 57 | 125 | 36 |
| Bristol ... ... ... | 10 | 3 | 156 | 97 | 273 | 127 | 78 | 23 | 19 | 10 | 55 | 27 |
| Cali ... ... ... | 70 | 59 | 97 | 120 | 184 | 148 | 20 | 19 | 42 | 33 | 129 | 33 |
| Caracas ... ... | 40 | 19 | 128 | 114 | 213 | 130 | 19 | 11 | 47 | 25 | 111 | 24 |
| Guatemala City ... | 59 | 45 | 98 | 110 | 106 | 101 | 23 | 13 | 70 | 49 | 169 | 20 |
| La Plata ... ... ... | 22 | 10 | 183 | 103 | 247 | 114 | 31 | 7 | 48 | 26 | 75 | 21 |
| Lima ... ... ... | 99 | 57 | 113 | 136 | 195 | 117 | 30 | 16 | 52 | 31 | 79 | 19 |
| Mexico City ... ... | 53 | 28 | 62 | 95 | 162 | 141 | 52 | 28 | 152 | 72 | 125 | 29 |
| Ribeirao Preto ... ... | 167 | 78 | 138 | 87 | 245 | 189 | 38 | 18 | 41 | 23 | 80 | 24 |
| San Francisco ... ... | 18 | 6 | 128 | 99 | 282 | 127 | 28 | 8 | 94 | 59 | 121 | 58 |
| Santiago ... ... | 107 | 34 | 128 | 121 | 210 | 148 | 82 | 33 | 194 | 83 | 157 | 32 |
| Sao Paulo ... ... | 43 | 20 | 103 | 96 | 256 | 191 | 43 | 19 | 47 | 20 | 64 | 20 |

an attempt to provide " a comprehensive account, as accurate and as compar- able as possible, of the causes of mortality of adults in highly diverse an widely separated populations" (See Table, p.3).

**The Bristol Survey**

It is not my intention to weary you with the origin, development an methodology of this large collaborative research project. Suffice it to say tha for us it meant setting up a research team of doctors, medico-social worker and clerk-typists, who investigated minutely about 2,000 deaths a year for tw years in the age group 15-74 years. The completeness of the data for Bristo was striking testimony to the willing co-operation of all concerned and I hav seized this opportunity, through the Long Fox Memorial Lecture, to place o record my grateful thanks and to acquaint you with some of the main finding It can be claimed from the results that Bristol has as high standards as an of the 12 cities in the survey in accuracy of death certification (based o *reliability* of *diagnostic criteria,* quality of medical service, and accessibilit to and use of services by members of the public). This is not to say tha certification practice in Bristol is by any means perfect as Dr. Alderson (o whom the brunt of the enquiry fell) showed in his M.D. thesis. In the firs year's sample of deaths, for example, 20% of the certificates required mino alteration of coding and 10% a major alteration. Hospital doctors (mostl young housemen) were more often " at fault " than G.P.'s; older patient certificates needed more frequent alteration; non-teaching hospitals neede more change than teaching hospitals. But this is not the purpose of my lectur and in any event would need a lecture in itself to do it justice. In the fina analysis 66% of the deaths (28,187) were in the same category of the 74 cause on the original and final assignments. By city the percentage of deaths remain ing in the same category ranged from 55 in Cali to 78 in Bristol.

**Broad Results of Survey**

It will be readily appreciated that the age and sex composition of nort Central and South American cities and Bristol all vary as do the enviro mental circumstances, cultures of the countries and customs of the people; s to effect true comparisons of the results various statistical techniques had to b employed to give age-adjusted and age-specific rates.

1. **Annual Age-Specific Death Rates for the 12 Cities** (Fig. 1)

From the point of view of death rates from all causes Bristol males und the age of 45 had the lowest age-specific mortality rates of any of t 12 cities investigated but thereafter the rates increased rapidly until the 65-74 age group they had the highest rate. On the other hand, Brist women retained the lowest position in all but the 65-74 age group. Brist males follow closely the mortality experience of England and Wales as whole and Bristol females have lower mortalities than the female rates f England and Wales except in the very high age groups.

The full report—" Patterns of Urban Mortality "—covers 353 pages inclu ing 18 appendices and clearly the most I can hope to do in this brief lecture to mention a few of the findings which have more relevance to Bristol.

Figure 1.

AGE-SPECIFIC MORTALITY RATES

DEATH RATES PER 100,000 ( ALL CAUSES )

by 10 year age groups

MALES

FEMALES

RANGE OF 11 OTHER CITIES

BRISTOL

10,000

1,000

100

Death rate per 100,000

15-24 25-34 35-44 45-54 55-64 65-74

Age group

2. **Infective and Parasitic Disease**

7.3% of the total deaths studied were due to these conditions. Bristol was the most favoured city (only 1.5% male death rate and 0.9% female rate). Mortality in Ribeirao Preto was 20 times as high as Bristol but in the former city *Chagas'* disease was the most serious problem whereas in all other cities it was *tuberculosis*.

3. **Cancer**

There was confirmatory evidence in nearly 90% of all the cancer deaths Mortality from cancer of all sites for males varied much more than fo[illegible] females for the 12 cities, e.g. the male rates for La Plata were three time[illegible] higher than those for Mexico City.

(i) **Alimentary-Canal**

An interesting feature emerged about cancer of the large intestine an[illegible] rectum. Mortality from left colonic cancers (sigmoid and rectum) was mor[illegible] variable than for right colonic cancers (i.e. " other colon "). Bristol, along wit[illegible] San Francisco and La Plata, showed higher rates for both, and for cancer o[illegible] the rectum in males Bristol had the highest rate of all cities. On the othe[illegible] hand, age adjusted death rates for stomach neoplasms were third lowest i[illegible] Bristol.

(ii) **Lung and Bronchus**

Male mortality was particularly high for lung and bronchus neoplasms i[illegible] Bristol and La Plata and the contrast between La Plata and other Lati[illegible] American cities is remarkable.

The full report includes a consideration of the epidemiological specificit[illegible] of different histological types of malignant disease arising in the lung, and o[illegible] a small sample of histological material from Bristol which when re-examine[illegible] at the National Cancer Institute of Bethesda raised the question of whethe[illegible] the rates of Groups I to Group II tumours are overstated in the U.K. a[illegible] compared with the U.S.A.

Compared with other cities La Plata had a high rate from cancer of th[illegible] urinary bladder in males. This city also had a high male rate from cancer [illegible] the larynx and lung and bronchus. By contrast Bristol with a high male ra[illegible] for lung and bronchus had low rates for larynx and bladder. Cigarett[illegible] smoking has been cited as an ætiological factor for all three sites but th[illegible] pattern for Bristol, La Plata and San Francisco cannot be explained by an[illegible] simple relationship.

(iii) **Breast and Cervix**

There was the expected inverse relationship between the levels of mortalit[illegible] from cancer of the cervix and of the breast in females. " In Bristol, La Pla[illegible] and San Francisco death rates from cancer of the breast were generall[illegible] higher throughout the age range than the rates for cancer of the cervix."

4. **Respiratory Disease** (Fig. 2)

The high death rate from bronchitis in Bristol males was striking and th[illegible] high mortality is not shown by Bristol females compared with females in oth[illegible] cities. Virtually all the Bristol deaths were from *chronic* bronchitis and th[illegible]

Figure 2.

DEATH RATES PER 100,000 from BRONCHITIS

(I.C.D. 500 - 502) by 10 year age groups.

excessive rate becomes apparent in the 45-54 age group and increases markedly in the 55-64 and 65-74 age groups.

" In Bristol a history of bronchitis was obtained for many deaths in which bronchitis was not assigned as a cause." (14.4% males and 8.3% females.) A careful study of individual records in San Francisco and Bristol showed that in the former the chronic respiratory disease was " dry " whereas in Bristol it was " wet " (i.e. with a productive cough) and no evidence was forthcoming that differences between these two cities was due to diagnostic or classification problems.

Bristol in particular, but also La Plata and Santiago displayed distinct seasonal variations in mortality from influenza, pneumonia and bronchitis and the same variations for arteriosclerotic heart disease. (Bristol has peaks in January-February and troughs in July.)

### 5. Cardiovascular Diseases

Cardiovascular diseases (comprising diseases of the heart and vessels) and also vascular lesions affecting the C.N.S., were the most frequent cause of death in nearly all cities. But there are marked differences, the rates being highest in San Francisco (males) and Bogota (females) and lowest for both sexes in Guatemala City.

In Bristol as in San Francisco, the high male rate is largely the result of the excessive mortality from arteriosclerotic and degenerative heart disease. The female populations of these two cities also have an increased tendency to die from these diseases but their mortality from cardiovascular conditions as a whole is less than average (C.M.F. for Bristol=79).

### 6. Diseases of the Digestive System

Much of the variation observed in death rates in this group is caused by differences in mortality from cirrhosis of the liver, especially when associated with alcoholism. It is evident that in three cities alcoholism is a significant public health problem.

Bristol residents of both sexes have the lowest death rates of all cities from diseases of the group as a whole and from cirrhosis with mention of alcoholism.

### 7. External Causes (Accidents, Poisonings and Violence)

Mortality rates from external causes also show more variation in men than in women.

Accidents are in general responsible for the major part of the death rate from external causes but in several cities, suicide and homicide are important causes of death in males and are by no means negligible in the women of San Francisco. In Bristol the female rate is unremarkable but the male rate is lower than that of any other city, to some extent because of the infrequency of deaths from homicide !

### 8. **Diabetes Mellitus**

Edward Long Fox's death was certified as due to diabetes mellitus of 7 years standing so perhaps I should say a few words about the findings for this condition.

The full report contains fascinating data and speculation. For example, using a formula devised by Moriyama and Chiang (1967) it is estimated that Bristol had 421 males aged 35-54 and 984 males aged 55-74 years during the years 1962-64 and 1784 and 2919 females in these two age groups. No one knows the true prevalence of diabetes mellitus in Bristol but this estimate gives 6108 cases in an effective population of 309,107 or 1 in 50 or 2% for both sexes combined.

Mexico City with an age-adjusted D.R. of 37.6 per 100,000 population was more than 8 times the lowest rate—in Bristol (4.4)—and analysis of the data showed this to be partly due to higher prevalence and partly to a higher case fatality rate in Mexico City than in Bristol.

" Persons known to have had diabetes were more likely in Mexico City than in Bristol to have died from the direct complications of the disease. In Bristol the deaths of diabetic subjects were more frequently assigned in whole or in part to arteriosclerotic heart disease."

" The higher prevalence in Mexico City may well have a genetic basis. Hitherto the highest prevalence for any population in the world is among the Pima Indians of Arizona, ethnically related to the indigenous population of Mexico."

It is also interesting to record that in a world wide study of congenital malformations Stevenson *et al.* (1966) commented upon the frequency of maternal diabetes in mothers confined at two large maternity hospitals in Mexico City.

" The three cities with the highest death rates from diabetes mellitus (Bogota, Guatemala City and Mexico City) are also the three cities situated at the highest altitudes. People living at high altitudes exhibit metabolic and structural adaptations to the lower oxygen tension." And, " lower blood glucose levels and flatter glucose tolerance curves in healthy adult males living at 4540 meters when compared with those living at sea level " have been noted. There is a clear case for further study of the effects of very high altitudes on diabetic subjects.

### **What conclusions can be drawn from the Survey?**

So far as I know this is the first investigation of comparative adult mortality which has been founded on such very complete information and analysed by standardised procedures.

Perhaps the cities of the developing countries have gained more new knowledge about their mortality problems than we in Bristol, but the surprising disparities in mortality for specific causes between cities highlights the possibility of reducing mortality in the productive period of life in each one of them.

Many of the deaths are from causes amenable to preventive action or treatment, e.g. infective and parasitic disease, alcoholism, external causes, bronchitis, and cancer of the bronchus. Most of the cities had a very favourable rate for at least one cause of death and " on the basis of the most favourable experience for this group of causes the total deaths would be reduced by 29% in males and 23% in females and the number of deaths of persons 15-34 years could be practically cut in half ".

But perhaps one of the most important aspects of the survey has been to identify with certainty populations at high and low risk—differences which can no longer be shrugged off by attributing them to differences in nosology or classification.

" Arteriosclerotic heart disease is clearly a much more serious threat to males in San Francisco and Bristol than in the Latin American cities " and " further epidemiological research is needed to determine the specific environmental and possible genetic factors responsible ".

There is an urgent need to find out why in La Plata the male age-adjusted death rates from cancer of the larynx, lung and bronchus, and urinary bladder were very high, whereas in Bristol although the rates for lung and bronchus are high those for the larynx and bladder were not high. Might it be that the differences in types of tobacco used in these cities is a possible reason?

" Bronchitis was the assigned cause for 11% of the deaths of males in Bristol and a history of bronchitis was given for 25% of all deaths of males " in our City. It is by no means so certain that cigarette smoking and atmospheric pollution account for these high rates.

For other cities there were equally unexplained differences in mortality which clearly merit further enquiries into social, environmental or genetic factors.

Apart from indications for preventive action and further epidemiological enquiry, the survey disclosed the need for development and improvement of standard procedures.

It is clear that " additional information is available in hospital and autopsy records which, combined with the clinical data, permit the causes of death to be defined more precisely ". " Completion of the medical certificate at the time of death in order that the burial permit may be issued inhibits search of hospital records or use of autopsy findings for additional information by the certifier." There is a need for " the development of systems for linking confidential information from autopsies and from hospitals with death certificates for the assignment of causes of death ".

There is also a need for the development of standard medical terminology which could be taught in all medical schools and " systems of recording and analysing multiple causes of deaths . . . require international standards, so that the comparable statistics will become available for future epidemiological studies."

In the last decade or so it has become fashionable to devalue mortality statistics as a yardstick of health progress and a basis of health planning. In fact, comparative mortality studies such as this, in which all Bristol doctors collaborated so generously, illustrate the increasingly important part they should play in the decades to come.

### SOURCES

Obituary, Edward Long Fox, M.D., OXON., F.R.C.P. *Brit. med. Jl.* April 5th, 1902.

Farr, W. (1839) In " First Annual Report of the Registrar General of Births, Deaths, and Mariages in England ". *H.M.S.O.*, p. 8.

W.H.O. Chronicle, Vol. 13, No. 6, 1959 " International Work in Health Statistics 1948-1958 ".

Patterns of Urban Mortality: A summary report of the Inter-American Investigation of Mortality. G. Wynne-Griffith, M.D., D.P.H., Ruth R. Puffer, Dr. P. H., *et al.* P.A.H.O. and P.A.S. Reg. Off. W.H.O., Washington, D.C., 1967.

Alderson, M. R. (1964) Unpublished M.D. thesis on " The accuracy of the Certification of Death and the classification of Underlying Cause of Death from the D.C."

*Acknowledgments* :

The Inter-American Investigation of Mortality is a research project of the Pan-American Health Organisation made possible by Research Grant GM-08682 of the National Institute of General Medical Sciences of the United States Public Health Service and by the co-operation and support of Ministries of Health, local health authorities and schools of medicine and public health in the twelve cities.

Due credit should be given to the local research team which in addition to myself as Principal Collaborator consisted of Dr. M. R. Alderson, M.D., B.S., D.C.H., D.P.H., D.R.C.O.G., full-time (now with University of Manchester, Department of Social and Preventive Medicine), Dr. K. E. Faulkner, M.B., Ch.B., D.P.H., D.C.H., part-time, Miss F. H. Chamberlain, S.R.N., S.C.M., Q.N., H.V., full-time (second health visitor) and Mrs. M. R. Livings, full-time secretary. My thanks are also due to Dr. P. N. Dixon, M.A., M.B., B.Chir., D.Obst.R.C.O.G., D.P.H., for his help with the preparation of some of the statistical material.

## Figures and Tables

**Figure 1. f1:**
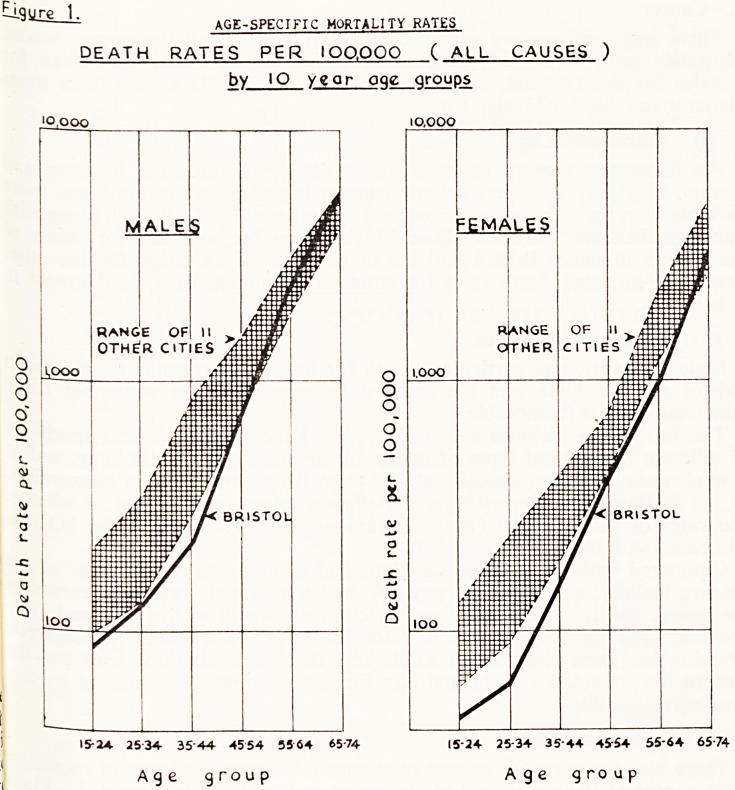


**Figure 2. f2:**